# Identification of additional dye tracers for measuring solid food intake and food preference via consumption-excretion in *Drosophila*

**DOI:** 10.1038/s41598-022-10252-6

**Published:** 2022-04-13

**Authors:** Brandon C. Shell, Mike Grotewiel

**Affiliations:** 1 Department of Human and Molecular Genetics, School of Medicine, Virginia Commonwealth University, Richmond, VA 23298 USA; 2 VCU Alcohol Research Center, Virginia Commonwealth University, Richmond, VA USA

**Keywords:** Biological techniques, Neuroscience, Physiology

## Abstract

The *Drosophila* model has become a leading platform for investigating mechanisms that drive feeding behavior and the effect of diet on physiological outputs. Several methods for tracking feeding behavior in flies have been developed. One method, consumption-excretion or Con-Ex, provides flies with media labeled with dye and then quantifies the amount of dye excreted into the vial as a measure of consumption. We previously found that Blue 1 and Orange 4 work well in Con-Ex and can be used as a dye pair in food preference studies. We have expanded our development of Con-Ex by identifying two additional dyes, Orange G and Yellow 10, that detect the anticipated effects of mating status, strain, starvation and nutrient concentration. Additionally, Orange G and Yellow 10 accumulate linearly in excretion products out to 48 h and the excreted volumes of these two dyes reflect the volumes consumed. Orange G also works with Blue 1 as a dye pair in food preference studies. Finally, consumption of Blue 1, Orange 4, Orange G or Yellow 10 does not affect ethanol sedation or rapid tolerance to ethanol. Our findings establish that Orange G and Yellow 10, like Blue 1 and Orange 4, are suitable for use in Con-Ex.

## Introduction

The *Drosophila* model is well-suited for investigating cellular-molecular mechanisms underlying feeding behavior and for exploring the role of diet on physiology or disease-like states (e.g.^1^). Flies have a powerful suite of genetic tools including existing mutants (e.g.^2^.), the GAL4-UAS system^3^ for tissue-specific RNAi^4^ and other transgene expression, and CRISPR for genome editing (e.g.^5^.). This suite of genetic tools allows investigators to manipulate the function of essentially any gene in the genome, many of which are conserved between flies and humans^6,7^. Additionally, the food media provided to flies is typically custom-made by investigators, allowing the composition of the media to be manipulated as an experimental variable^8^. These features of the *Drosophila* model have led to numerous advances including identification of serotonin receptors in metabolic processing^9^, elucidating a role for dietary yeast in ethanol sedation^10^, and description of neural structures that regulate hunger^11^. Importantly, these are but a few examples of the studies in laboratories using the *Drosophila* model to investigate feeding behavior and the effects of diet on key health-related outcomes.

Investigators have used a number of methods to assess food consumption in flies^12^, often a key aspect of studies on dietary effects or feeding behavior. For example, capillary feeding (CAFE) is used to measure liquid media consumption and media preference^13^ and Fly Liquid Food Interaction Counter (FLIC) is used to measure fly interactions with liquid food media^14^. *Drosophila* are maintained on agar-based food media under the standard housing conditions, and consequently methods using oligonucleotide^15^, radioactive^16^ and dye^17–19^ tracers to monitor solid media consumption have been developed. Furthermore the oligonucleotide^15^ and dye^17^ tracer methods have been further adapted to assess food preference. All of the tracer methods allow for the monitoring of media consumption, with the oligonucleotide^15^ and radioactive^16^ methods measuring the tracer remaining in the fly and the dye methods^17–19^ quantifying the amount of consumed tracer excreted from the fly.

In this report, we expand our dye-based consumption-excretion (Con-Ex) method^17,18^ for measuring solid food intake in flies. We previously characterized Blue 1 and Orange 4 as dye tracers suitable for Con-Ex. The studies described here identified two additional dyes (Orange G and Yellow 10) that also work well in Con-Ex, and found that Orange G and Blue 1 work as a dye pair in food preference studies as we previously reported for Orange 4 and Blue 1^17^. This report, in conjunction with our previous findings^17,18^, expand the dye options for use in Con-Ex, provide the opportunity to use multiple dyes to confirm or assess fly feeding behavior, and establish two dye pairs for assessing food preference in flies.

## Results and discussion

In our previous studies that identified Orange 4 as a dye tracer for Con-Ex and as a dye that could be paired with Blue 1 in Con-Ex food preference analyses^17^, males consumed more solid media than females when the media were labeled with Yellow 10, Yellow 6 and Light Green SF^17^. Greater consumption in males compared to females when tracked with these three dyes was somewhat unexpected based on previous studies using radioactive tracers to measure solid media consumption^16^ and our previous studies using Blue 1^18^ and Orange 4^17^ in Con-Ex. Nevertheless, our initial studies with these three dyes^17^ suggested they might have utility for Con-Ex. Additionally, although we did not fully explore them as food tracers, our initial characterization of Orange G and Cochineal Red A (hereafter Red A) suggested they might be promising candidate dyes for Con-Ex^17^. Finally, we also identified Green 5 as a possible candidate dye for Con-Ex given that it is water soluble and is not considered hazardous (spectrumchemical.com). We reasoned that additional studies with these six dyes might validate individual tracers for use in Con-Ex and also might identify pairs of tracers for Con-Ex food preference analyses. As in our previous studies^17,18^, we quantified ExVial (the amount of dye excreted into the vial) in the studies reported here because it is the best single measure of consumption in Con-Ex experiments. Dyes were used at 1% w/v unless noted otherwise.

**Figure 1:**
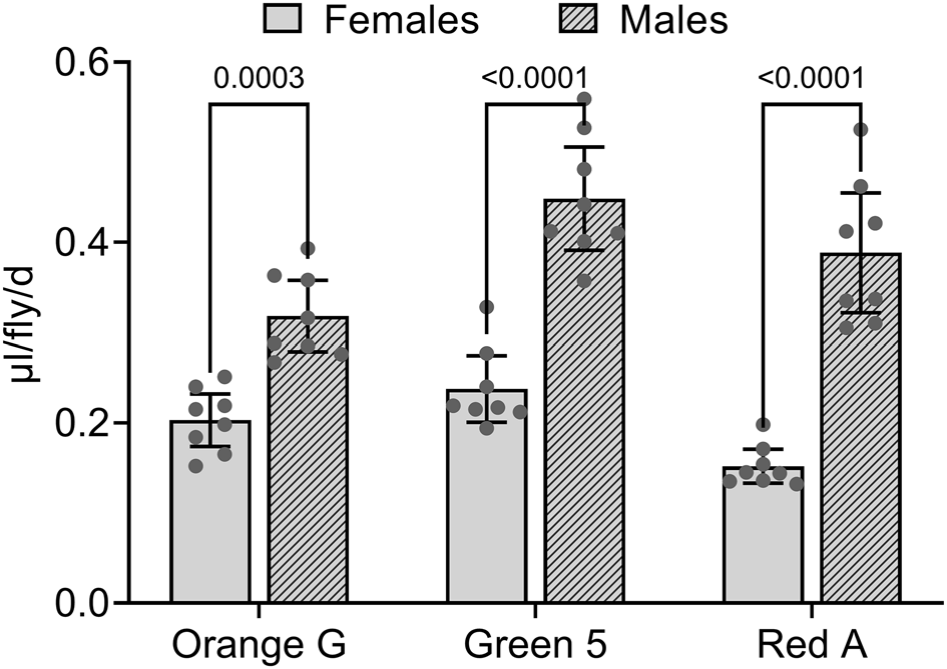
Con-Ex in control females and males determined with Orange G, Green 5 and Red A. GL flies consumed-excreted 2Y10S3C media containing the indicated dyes (1% w/v) for 24 h. Dye and sex significantly affected Con-Ex measured with all three dyes (two-way ANOVA; dye, *p* < 0.0001; sex, *p* < 0.0001; interaction, *p* = 0.0060; n = 8). Con-Ex with males was greater than females when measured with all three dyes (Bonferroni’s, *p* values shown for pair-wise comparisons).

We previously found that males consumed more solid food than females when the food media were labeled with Yellow 10, Yellow 6 and Light Green SF^17^. To determine whether males likewise consumed more solid food than females when the food media were labeled with Orange G, Green 5 and Red A, we performed 24 h Con-Ex experiments with each dye in our standard food medium (2Y10S3C) using GL males and females (Fig. 1). Males consumed more than females when the media were labeled with Orange G, Green 5 or Red A (Fig. 1). This dye by sex interaction, exemplified by females consuming more than males when media are labeled with Blue 1 and Orange 4^17,18^ and males consuming more than females when media are labeled with Yellow 10, Yellow 6, Light Green SF, Orange G, Green 5 and Red A^17^ and Fig. 1), calls into question whether males or female flies consume more solid media and whether Con-Ex with these dye tracers is suitable for investigating sexual dimorphisms in solid media consumption. Given the plethora of possible factors (media composition, duration of media consumption, effects of genotype, prior media exposure, etc.) that could influence the dye by sex interaction we observed, we opted to not explore this issue further. Instead, we proceeded to characterize the utility of Yellow 10, Yellow 6, Light Green SF, Orange G, Green 5 and Red A for tracking consumption of solid food media within each sex separately. We focused on females in some experiments because the effects of mating and starvation on solid media consumption are robust and reproducible in this sex^16–18^.

**Figure 2:**
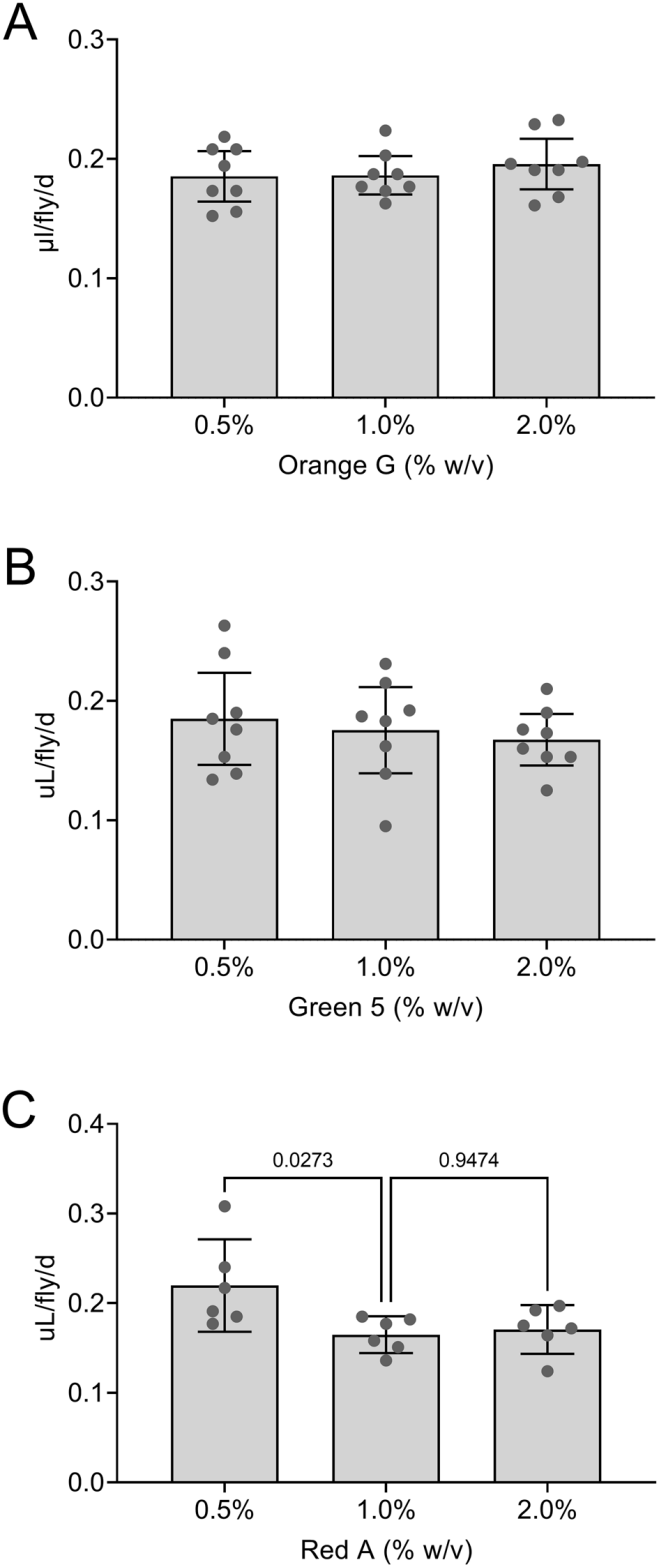
Effect of dye concentration on Con-Ex determined with Orange G, Green 5 and Red A in control females. GL females were allowed to consume-excrete 2Y10S3C media containing the indicated dyes at the concentrations shown for 24 h. (**A**,**B**) The concentration of Orange G (**A**) and Green 5 (**B**) did not significantly affect Con-Ex (individual one-way ANOVAs; Orange G, *p* = 0.4689; Green 5, *p* = 0.3937; n = 8). (**C**) The concentration of Red A significantly affected Con-Ex (one-way ANOVA, *p* = 0.0261, n = 6). Con-Ex determined using 0.5% and 1.0% Red A was significantly different (Bonferroni’s, *p* values shown for pair-wise comparisons).

We previously found that Blue 1^18^, Orange 4, Yellow 6, Yellow 10 and Light Green SF^17^ do not have major effects on the volume of food media consumed-excreted when used at concentrations that allowed these dye tracers to be readily detected in ExVial samples. To determine if Orange G, Green 5 and Red A impacted food media consumption-excretion, we assessed Con-Ex in control females provided with standard food media containing increasing concentrations of each dye. Twenty-four h Con-Ex was not affected by the concentration of Orange G (Fig. 2A) or Green 5 (Fig. 2B) at 0.5–2.0%. In contrast, Con-Ex decreased as the concentration of Red A was increased (Fig. 2C), suggesting that higher concentrations of this dye might suppress consumption. Based on these data, we further explored Orange G and Green 5 in this project, but did not further examine Red A.

**Figure 3:**
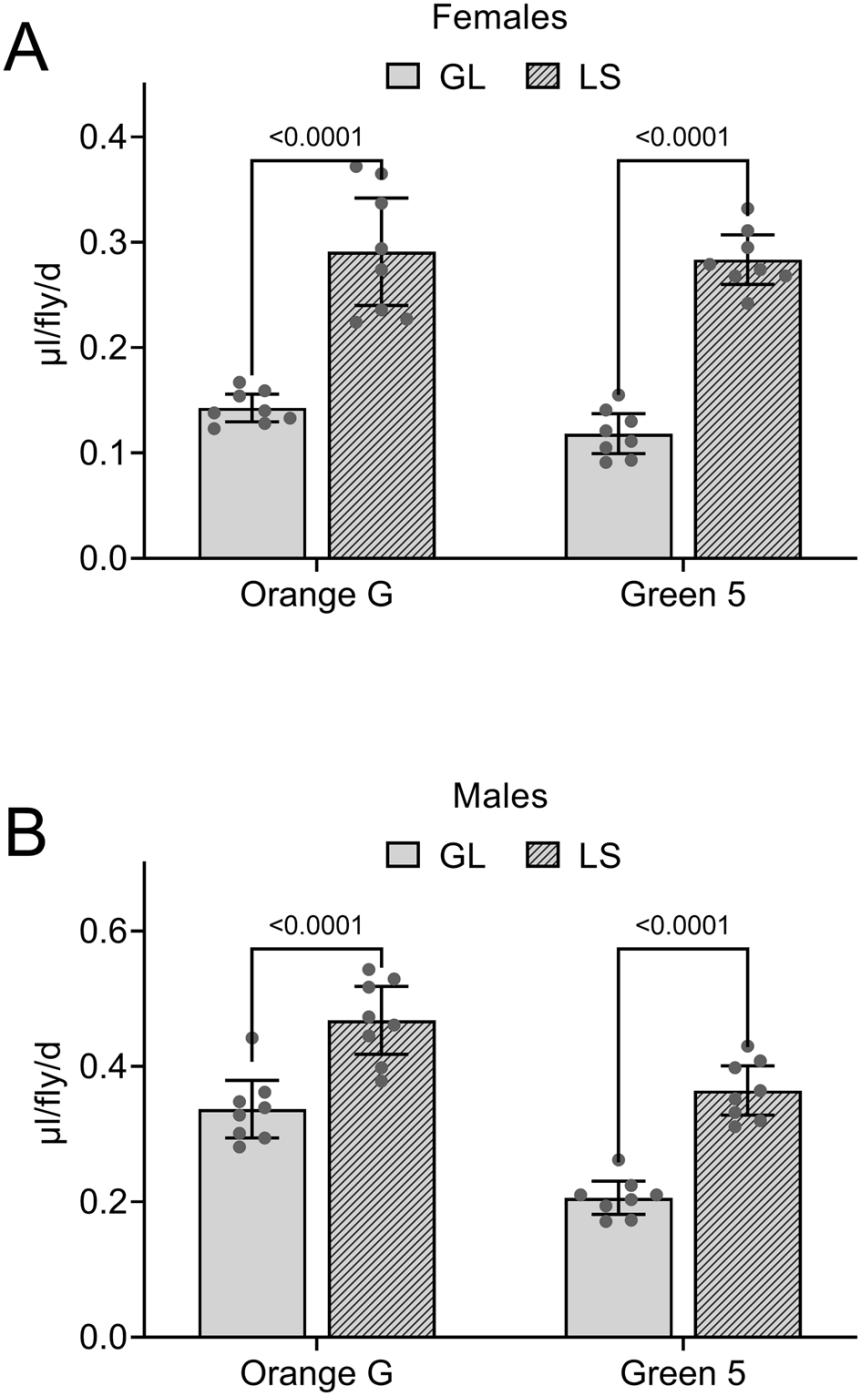
Effect of strain on Con-Ex determined with Orange G and Green 5. Flies consumed-excreted 2Y10S3C media with the indicated dyes for 24 h. (**A**) In GL and LS females, Con-Ex was significantly affected by strain, but not dye (two-way ANOVA; sex, *p* < 0.0001; dye, *p* = 0.2249; interaction, *p* = 0.5166; n = 8). (**B**) In GL males, Con-Ex was affected by strain and dye (two-way ANOVA; sex, *p* < 0.0001; dye, *p* < 0.0001; interaction, *p* = 0.4206; n = 8). Con-Ex was greater in LS compared to GL flies in all cases (Bonferroni’s, *p* values shown for pair-wise comparisons).

**Figure 4:**
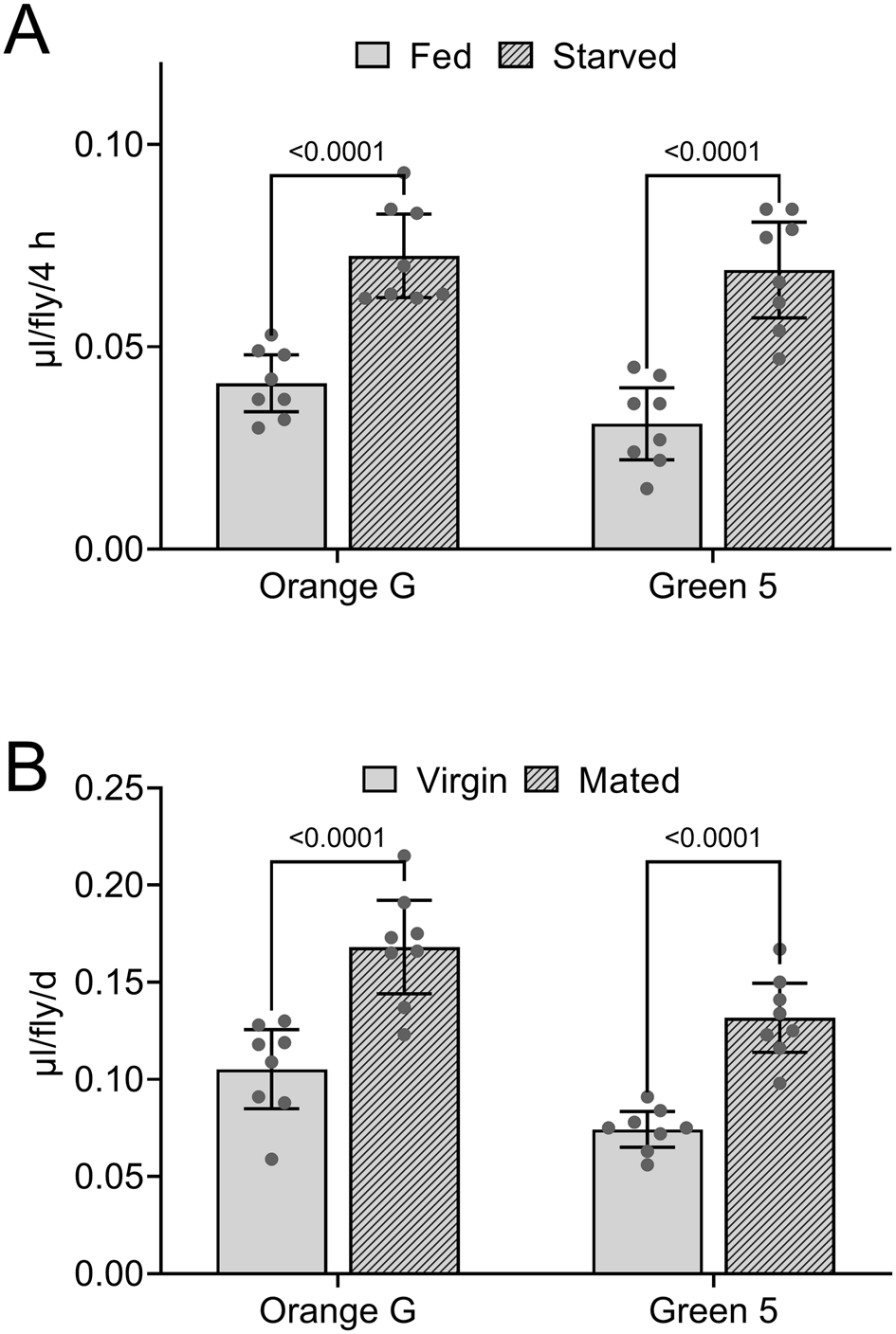
Effect of starvation and mating status on Con-Ex determined with Orange G and Green 5. (**A**) Starvation (16 h on 1% agar) significantly affected 4 h consumption-excretion of 2Y10S3C media labeled with Orange G and Green 5 (1% w/v) in GL females (two-way ANOVA; starvation, *p* < 0.0001; dye, *p* = 0.1102; interaction, *p* = 0.6097; n = 8). Con-Ex determined with both dyes was greater in starved flies (Bonferroni’s, *p* values shown for pair-wise comparisons). (**B**) Mating status and dye significantly affected 24 h consumption-excretion of 2Y10S3C media labeled with Orange G and Green 5 (1% w/v) in GL females (two-way ANOVA; mating status, *p* < 0.0001; dye, *p* = 0.0002; interaction, *p* = 0.7362; n = 8). Con-Ex with both dyes was greater in mated flies (Bonferroni’s, *p* values shown for pair-wise comparisons).

Blue 1, Orange 4, Yellow 6, Yellow 10 and Light Green SF exhibit many features supporting their utility as dye tracers for consumption-excretion of solid food media. These five dyes detect the predicted effects of strain, starvation and mating status^17,18^. We therefore determined whether Orange G and Green 5 shared these key features. As expected, Lausanne-S (LS) females (Fig. 3A) and males (Fig. 3B) consumed-excreted more solid food media than GL flies (the standard Grotewiel laboratory strain) when provided with food labeled with Orange G or Green 5. Also as expected, prior starvation in females increased Con-Ex (Fig. 4A) and mated females consumed-excreted more solid media than did virgin females (Fig. 4B) when measured with Orange G and Green 5 as dye tracers. Con-Ex with Orange G and Green 5 as dye tracers therefore detects the predicted effects of strain, starvation and mating status as found when using Blue 1, Orange 4, Yellow 6, Yellow 10 and Light Green SF as dye tracers^17,18^.

**Figure 5:**
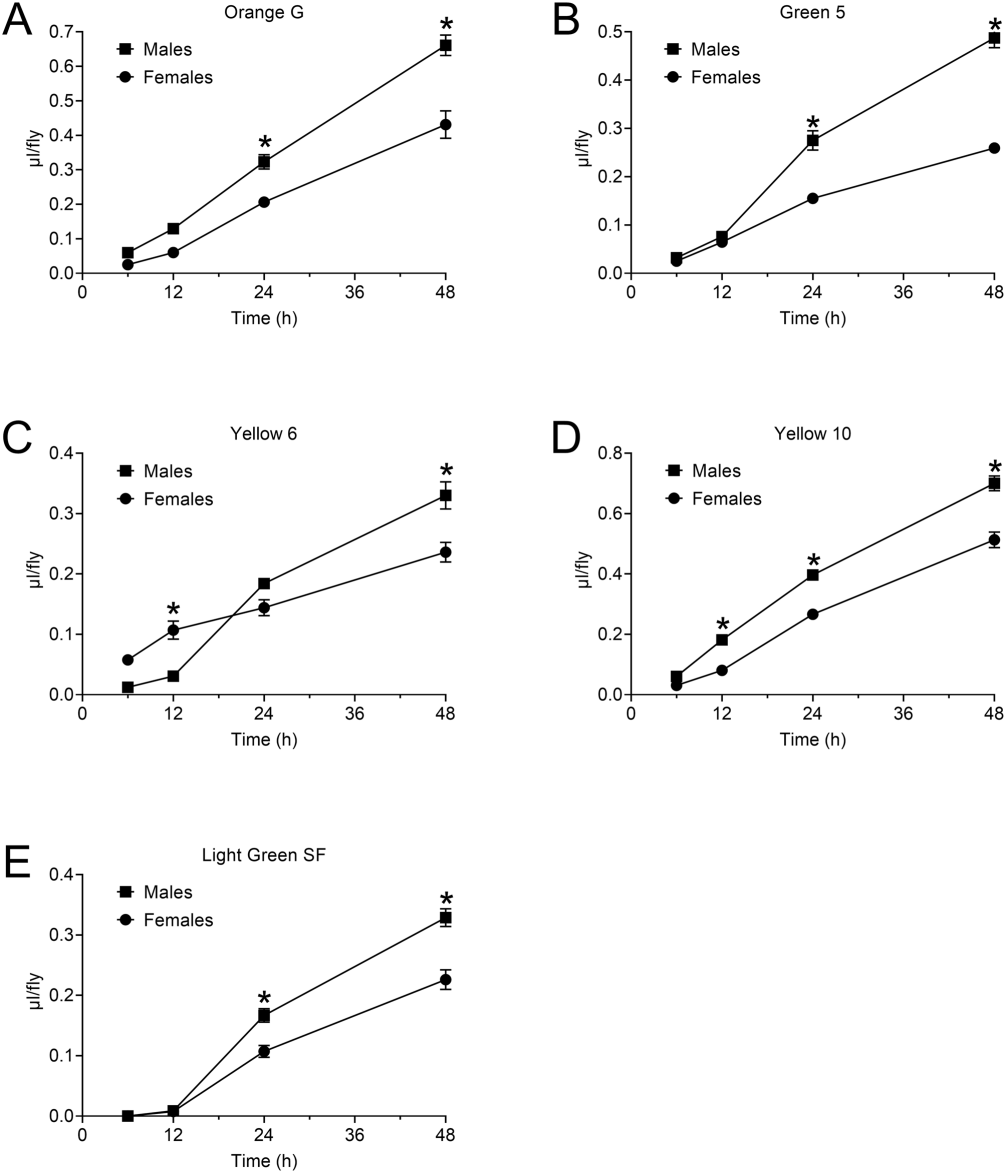
Con-Ex time-courses in control females and males with Orange G, Green 5, Yellow 6, Yellow 10 and Light Green SF. GL flies and 1% w/v dyes in 2Y10S3C media used in all studies. (**A**–**E**) Time affected Con-Ex measured with all five indicated dyes (individual two-way ANOVAs, *p* < 0.0001, n = 8). (**A**,**B**,**D**,**E**) Sex affected Con-Ex measured with Orange G, Green 5, Yellow 10 and Light Green SF (individual two-way ANOVAs, *p* < 0.0001). (**C**) Sex did not affect Con-Ex determined with Yellow 6 (two-way ANOVA, *p* = 0.7247). (**A**–**E**) There were interactions between time and sex in all experiments (individual two-way ANOVAs, *p* < 0.0001). Con-Ex was significantly different in females and males at the indicated time-points (*Bonferroni’s, *p* = 0.0005 to < 0.0001). Data are mean ± S.E.M.

Blue 1 and Orange 4 accumulate in a largely linear fashion in ExVial samples as the duration of exposure to dyed food media increases^17,18^. We explored whether Orange G, Green 5, Yellow 6, Yellow 10 and Light Green SF shared this feature of dyes suitable for Con-Ex. Orange G (Fig. 5A) and Yellow 10 (Fig. 5D) accumulated in ExVial as a simple function of feeding duration in control females and males. Additionally, males consistently consumed-excreted more dye at time-points where reasonable differences could be detected. Con-Ex measured with the other three dyes displayed temporal dynamics or had sex-specific effects that might limit their usage. Males did not consistently accumulate more Green 5 (Fig. 5B) and Yellow 6 (Fig. 5C) than did females across time, and accumulation of Light Green SF was not detectable until after 12 h of feeding in both males and females (Fig. 5E). Although additional studies are needed to fully understand the temporal and sex-specific dynamics of using these three dyes, the relatively simple time-dependent accumulation of Orange G (Fig. 5A) and Yellow 10 (Fig. 5D) in both males and females led us to focus these two tracers. Additionally, the differences in absorbance spectra of Orange G and Yellow 10 (Fig. S3A) suggested these two dyes might be suitable as partners with Blue 1 in food preference analyses (see below).

**Figure 6:**
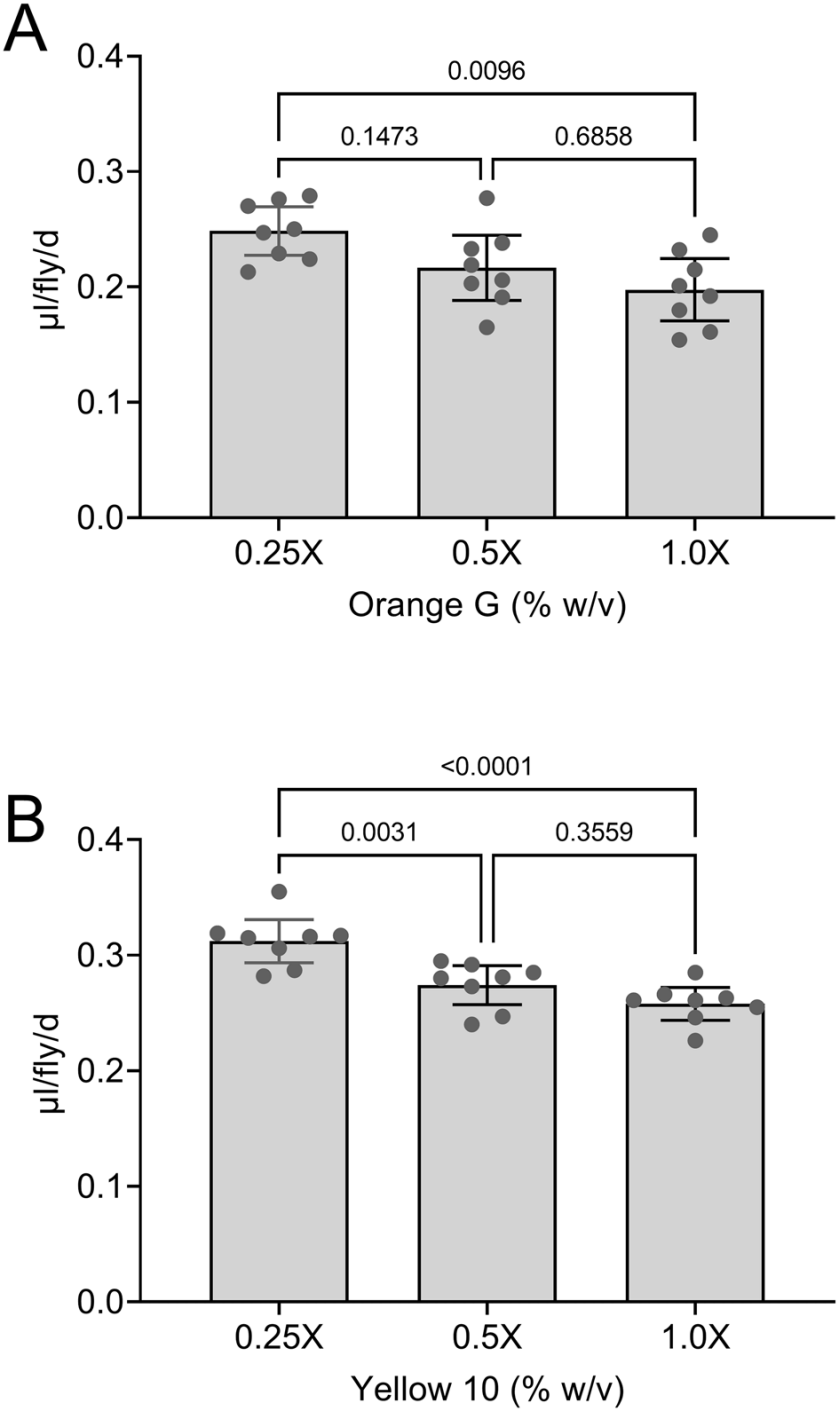
Compensatory feeding measured with Con-Ex using Orange G and Yellow 10. Con-Ex (24 h) in GL females measured with Orange G (**A**) and Yellow 10 (**B**) increased as the concentration of food media decreased (individual one-way ANOVAs; panel A, *p* = 0.0108, n = 8; panel B, *p* < 0.0001, n = 8). (**A**) Con-Ex with Orange G was significantly greater with 0.25X versus 1.0X media (Bonferroni’s, *p* values shown for pair-wise comparisons). (**B**) Con-Ex determined with Yellow 10 using 0.25X medium was significantly greater than with 0.5X and 1.0X media (Bonferroni’s, *p* values shown for pair-wise comparisons). 1.0X media is 2Y10S3C (see Material and Methods).

Flies decrease the volume of media they consume as the concentration of nutrients in the media increases^16–18^. We assessed whether Con-Ex with Orange G and Green 5 could detect this compensatory feeding. The volume of solid media consumed-excreted measured with both Orange G (Fig. 6A) and Green 5 (Fig. 6B) was higher when flies were provided a relatively low nutrient medium (0.25-fold of normal) compared to 1.0-fold medium. Con-Ex with Orange G and Yellow 10 is therefore capable of detecting consumption in response to manipulation of media nutrient concentration like we previously found for Con-Ex using Blue 1^18^ and Orange 4^17^.

**Figure 7:**
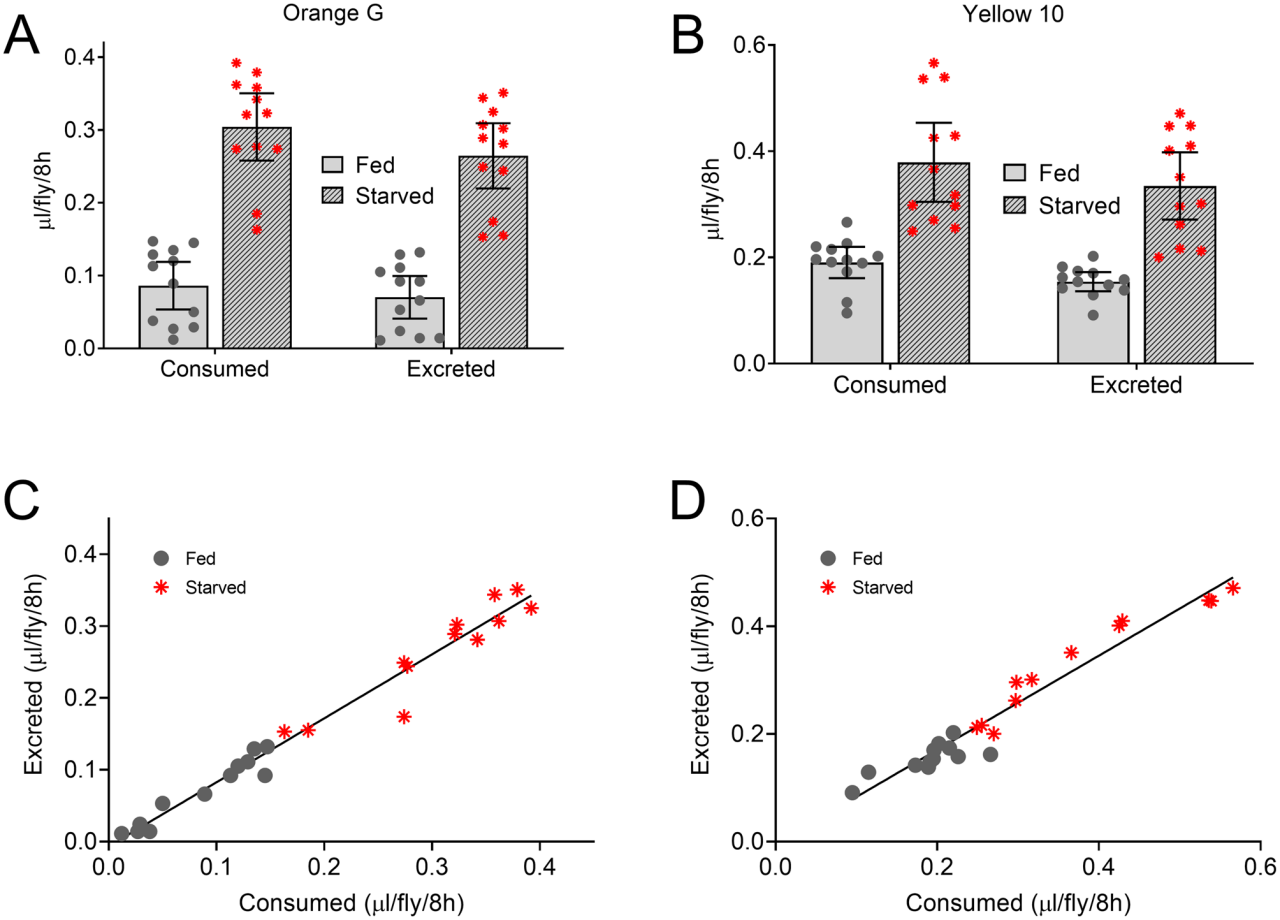
Coupled CAFE:Con-Ex studies with Orange G and Yellow 10. (**A**,**B**) Starvation, but not the method used to measure dye volume (i.e. CAFE consumed vs ExVial excreted), affected the volume of media determined with Orange G (**A**) and Yellow 10 (**B**) (individual two-way ANOVAs; starvation, *p* < 0.0001; consumed vs excreted, *p* = 0.0951 to 0.1238; n = 12). (**C**,**D**) The volume of media consumed via CAFE correlated with the volume of dye excreted via Con-Ex determined with Orange G (**C**) and Yellow 10 (**D**) (Pearson; Orange G, R^2^ = 0.9771, *p* < 0.0001; Yellow 10, R^2^ = 0.9472, *p* < 0.0001).

To determine whether the excreted volumes of Orange G and Yellow 10 reflect the volumes of dye (and therefore media) consumed, we performed coupled CAFE:Con-Ex studies^17,18^. Flies consumed dyed liquid media (measured via CAFE^13^) and then excreted dye into vials (measured as ExVial). We used fed and starved flies to generate a range of volumes consumed and excreted for correlation analyses. When using either Orange G or Yellow 10, starved flies consumed and excreted more dye than fed flies (Fig. 7A,B). The volumes consumed (measured via CAFE) and excreted (measured as ExVial) were indistinguishable when tracked by Orange G (Fig. 7A) and Yellow 10 (Fig. 7B). Additionally, the volumes consumed strongly correlated with the volumes excreted for groups of flies within each vial when tracked by Orange G (Fig. 7C) and Yellow 10 (Fig. 7D). These results indicate that the volume of Orange G and Yellow 10 excreted reflect, and could be equivalent to, the volume of Orange G and Yellow 10 consumed.

Statistical power (i.e. the ability to detect differences between groups) is an important consideration for any experimental method^20^. Using the average means and standard deviations from dozens of experiments, we found that the statistical power of Con-Ex using Orange G and Yellow 10 was comparable to that of Con-Ex with Blue 1 and Orange 4 (Fig. S2). For example, detection of 20% differences in ExVial values using Blue 1^18^, Orange 4^17^, Yellow 10 and Orange G is predicted to require 11, 12, 5 and 10 replicates, respectively. The number of replicates required to detect meaningful differences between groups is therefore reasonable in Con-Ex with all four dyes.

We previously found that Blue 1 and Orange 4 could be used as a dye pair in Con-Ex food preference studies^17^. One key feature of these two dyes is that their absorbance spectra do not overlap substantially, allowing each dye to be detected independently in ExVial samples that contain both dyes^17^. To explore whether Orange G and Yellow 10 could likewise be used in preference studies, we compared the absorbance spectra of these two dyes with Orange 4 and Blue 1. The spectra for Orange G and Blue 1 did not overlap substantially (Fig. S3A), suggesting that absorbance of neither dye would interfere with absorbance of the other in mixed samples. The absorbance spectra for Yellow 10 overlapped somewhat that of Blue 1 (Fig. S3A), making us cautiously optimistic that these two dyes might not significantly interfere with detection of the other. All other possible dye pairs had substantially overlapping absorbance spectra (Fig. S3A) and were not considered further.

We explored the possibility that Orange G and Blue 1 could be detected independently in mixed samples and that Yellow 10 and Blue 1 could likewise be detected independently. Blue 1 did not alter detection of Orange G and Orange G did not alter detection of Blue 1 (Figs. S3B and S3C), indicating that these two dyes can be measured independently in the same samples. Unfortunately, although Yellow 10 did not interfere with detection of Blue 1 (Fig. S3E), Blue 1 interfered with detection of Yellow 10 (Fig. S3D) in mixed samples, precluding the use of Yellow 10 and Blue 1 as a dye pair for Con-Ex food preference experiments.

**Figure 8:**
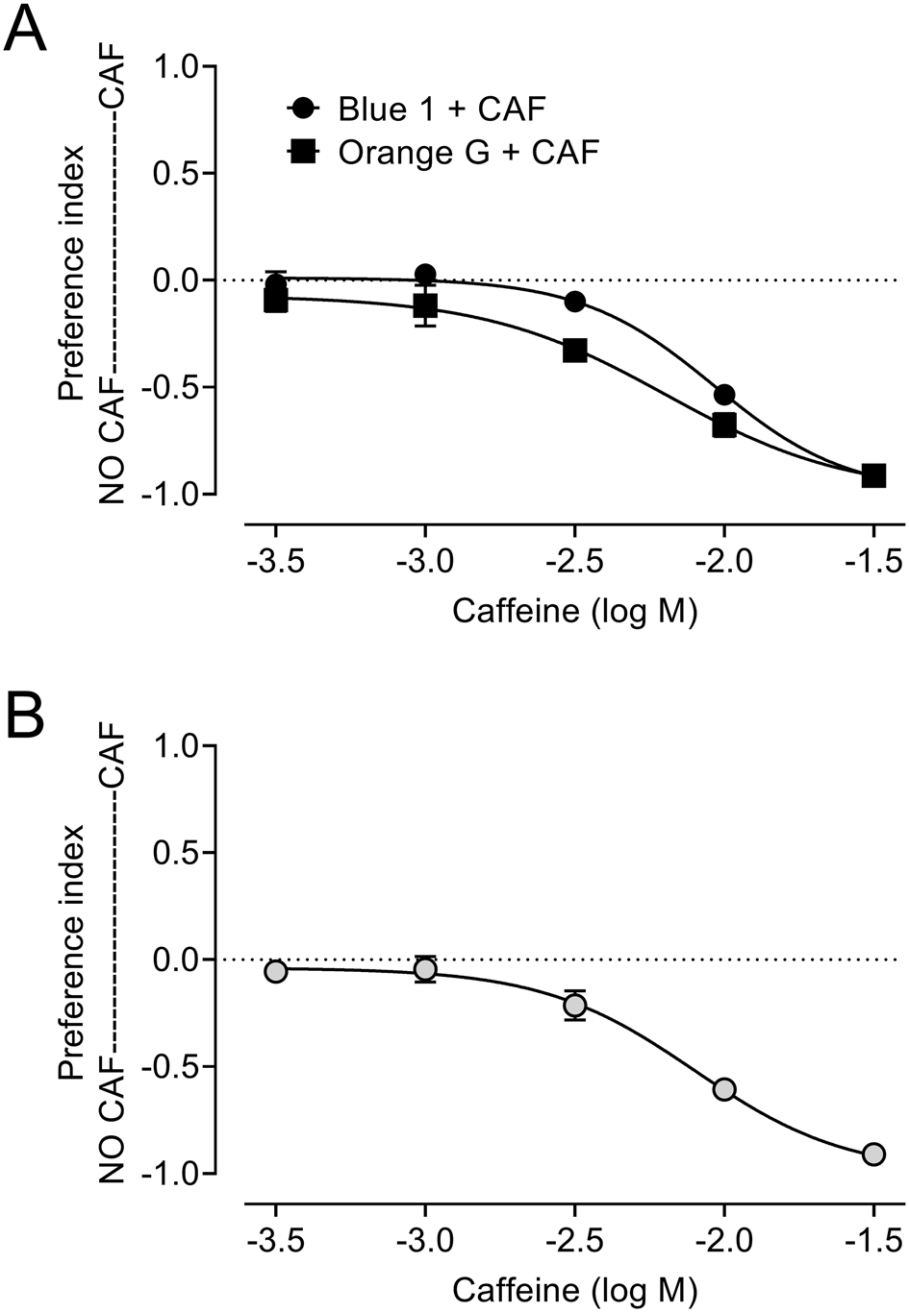
Food preference measured with Blue 1 and Orange G in response to caffeine. GL females and 5Y10S media were used in all studies. Dyes were used at 1% w/v. Flies were allowed to consume-excrete media for 24 h. (**A**) Preference index determined with medium labeled with Orange G vs medium labeled with Blue 1 containing the indicated concentrations of caffeine (circles, n = 8) or medium labeled with Blue 1 vs medium labeled with Orange G containing the indicated concentrations of caffeine (squares, n = 8). (**B**) Preference index compiled from data in panel A (n = 16, IC_50_ = 7.97 mM). Curves are least squares fit from non-linear regression (log[caffeine] vs preference index, bottom constrained to − 1; r^2^ = 0.9322 to 0.9608).

To test whether Blue 1 and Orange G could serve as a dye pair in food preference studies, we provided flies with a divided feeder cap^17^ containing food media labeled with Blue 1 in one-half of the cap and Orange G in the other half of the cap. We added increasing concentrations of caffeine, an aversive tastant^17,21,22^, to the media labeled with Blue 1 in one set of experiments and added increasing concentrations of caffeine to the media labelled with Orange G in a parallel set of experiments. We allowed flies to consume the media labeled with Blue 1 or Orange G for 24 h, then measured Blue 1 and Orange G in the resulting ExVial samples. Increasing concentrations of caffeine in either food labeled with Blue 1 or food labeled with Orange G made flies prefer the media without caffeine (Fig. 8A). Combining these reciprocal data sets, we found that caffeine led to a dose-dependent preference of unadulterated food media (Fig. 8B). The IC_50_ (inhibitory concentration 50, the concentration required to change the preference index to − 0.5) in the studies described here with Blue 1 and Orange G as a dye pair was 7.97 mM, which is nearly identical to the IC_50_ for caffeine obtained in a separate set of experiments using Blue 1 and Orange 4 as a dye pair (7.98 mM^17^). The dye pairs Blue 1-Orange G and Blue 1-Orange 4 therefore both appear to work well and provide comparable results in Con-Ex food preference studies with aversive tastants.

We initially became interested in developing dye-based solid media consumption methods through observing that yeast, a standard component of fly media, has a substantial impact on ethanol sedation in flies^10^. In this previous study, we showed that consumption of Blue 1 did not affect ethanol sedation. Here, we extended these previous experiments to address whether consumption of Blue 1, Orange 4, Orange G and Yellow 10 for 24 h altered ethanol sedation or rapid tolerance to ethanol. Ethanol sedation (Fig. S4A) and rapid tolerance (Fig. S4B) were not discernably affected by providing flies with any of the four dye tracers in standard media for 24 h. These four dye tracers should, therefore, be suitable for determining the relative consumption of drugs presented to flies on solid food media in studies assessing the effects of pharmacological agents on these two behavioral responses to acute alcohol.

### Summary

The data described here, in conjunction with our previous studies^17,18^, establish Blue 1, Orange 4, Orange G and Yellow 10 as dyes suitable for measuring solid food consumption in Con-Ex studies. Con-Ex measured with these dyes can detect the predicted effects of strain, starvation, mating status, and media nutrient concentration. Two pairs of these dyes (Blue 1-Orange 4 and Blue 1-Orange G) can be used in preference studies with Con-Ex, and at least one of these dye pairs can be adapted for preference studies with EX-Q, a consumption method related to Con-Ex^19^. As described previously^17,18^, advantages of using Con-Ex to measure food media consumption with dye tracers include low cost, straightforward assay protocols, ability to use essentially standard housing conditions with solid agar-based media, and the ability to adapt the method for measuring food preference. Other methods for measuring solid food intake in *Drosophila* use radioactive tracers^16^ or oligonucleotide tracking^15^. These methods are sensitive and have high statistical power. It is possible, though, that the use of radioactive tracers might not be feasible in all laboratory settings and that the protocols for detecting oligonucleotides might be cost prohibitive for large-scale laboratory usage in some cases. Additionally, it might be challenging to identify radioactive tracers with distinct emission characteristics such that they are suitable for food preference studies. The decision to use dye, radioactive or oligonucleotide tracers to assess solid food intake in *Drosophila* will likely be driven by practical considerations including the anticipated cost of the experiments, ease of performing the studies, and statistical power of the method.

Regardless of the method used to assess solid food intake, we envision several major applications for them. One application will be measuring nutrient intake in flies provided with different diets. These studies will determine whether altered nutrient intake in flies is truly influencing disease-like physiological states as expected, thereby potentially eliminating the possibility that some change other than altered nutrient consumption is driving the phenotypes being investigated. Another application of these methods will be to assess whether drugs included in the food media are consumed by flies and, if so, the amounts consumed. Such studies will also allow investigators to formally exclude (or further consider) the possibility that the drugs are altering nutrient consumption and in turn the altered food consumption is impacting the fly phenotype being investigated. Another application of methods for measuring solid food intake in flies will be to explore genetic and neurobiological mechanisms underlying food intake and food preference. A better understanding of the mechanisms driving food consumption and preference could have a profound, positive impact on our ability to mitigate a large number of diet-related diseases including obesity, type 2 diabetes, and several forms of cancer.

## Materials and methods

### Fly stocks and husbandry

A standard fly medium (2% yeast, 10% sucrose, 3% cornmeal, and 1% agar; hereafter 2Y10S3C) supplemented with antibiotics (ampicillin, chloramphenicol, and tetracycline; Sigma-Aldrich, St. Louis, MO, USA) and an anti-fungal agent (tegosept; Sigma-Aldrich, St. Louis, MO, USA) was used to rear all flies to adulthood as described^17,18^. Flies were grown in an environmental chamber at 25 °C and 60% relative humidity with a 12:12 h light/dark cycle (lights on 7:00 a.m.-7:00 p.m.). GL, the standard control strain used in Grotewiel laboratory harbors the *w*^+^ allele from Canton-S in an isogenic background as previously described^17^. Lausanne-S (LS) flies were obtained from the Bloomington *Drosophila* Stock Center (#4268; Bloomington, IN, USA).

### Materials

Propylene bottles (Fisher Scientific, AS-355; Waltham, MA, USA) and polystyrene vials (VWR, 75813-160; Radnor, PA, USA) were used, respectively, to rear flies and to assess Con-Ex and ethanol sedation. Dyes used were previously reported^17,18^ except for Green 5 (Spectrum Chemical, New Brunswick, NJ, USA). Dyes can have multiple common names and therefore suppliers, product numbers and CAS numbers for all dyes used in these studies are provided in the Supplementary Table. All dyes were used at 1% w/v unless indicated otherwise. Absorbance of each dye was determined at the following wavelengths (in parentheses): Yellow 10 (412 nm), Orange G (475 nm), Yellow 6 (485 nm), Red A (506 nm), Green 5 (608 nm), Light Green SF (630 nm) and Blue 1 (630 nm). A Pharmacia Biotech spectrophotometer (Ultraspec 2000) was used with polystyrene disposable cuvettes (Fisher Scientific, 14-955-127; Waltham, MA, USA) with a 1 cm light path to measure the absorbance values. Caffeine was purchased from Sigma-Aldrich (St. Louis, MO, USA). Ethanol, flugs and silicone plugs used to assess ethanol sedation and rapid tolerance were as described^23,24^. Capillary tubes for coupled CAFE:Con-Ex studies were from VWR Scientific (borosilicate glass micropipets, 5 uL, 53432-706; Radnor, PA, USA) as described^17,18^.

### Consumption-excretion analyses

Studies were performed essentially as described^17,18^. Fourteen to 15 days after initial seeding of bottles, flies were collected between 10:00 and 11:00 am via brief CO_2_ anesthesia with CO_2_ pads (Genesee Scientific; San Diego, CA, USA) and stereo microscopes (Zeiss Stemi 2000-C; Oberkochen, Germany). Anesthetized flies were sorted by sex into groups of 15 and placed in empty vials. A cap with dyed-food media was placed on top to initiate experiments. Dyes were used at 1% w/v unless otherwise noted. Food media were 2Y10S3C (2% yeast, 10% sucrose, 3% cornmeal, and 1% agar as defined above) in all studies except those on preference, which used 10% sucrose-5% yeast (10S5Y) to be consistent with our previous preference experiments^17^. For preference studies, there was no discernable preference for media labeled with 1% w/v Orange G compared to media labeled with 1% w/v Blue 1 in the absence of the aversive tastant caffeine (preference index =  − 0.0791 ± 0.05211 (mean ± S.E.M.), n = 8, one-sample t test compared to theoretical mean of 0, *p* = 0.1732, t(7) = 1.1516).

All studies were performed under a 12:12 h light:dark cycle at 25 °C/60% relative humidity. Flies were allowed to consume the dyed-media and excrete dyed waste products inside the vial (ExVial) for the amount of time indicated (typically 24 h, but see figure legends). ExVial samples were collected in 3 mL water or phosphate-buffered saline (PHB, pH 7.6) and the concentrations of dye along with the volumes of excreted media were determined as described^17,18^. Standard curves, ExVial absorbances and ExVial volumes were not different when Orange G and Yellow 10 (two main dye tracers examined here) were in water versus PBS (Fig. S1). Starvation when used was achieved by housing flies for 18 h on agar-only food.

Absorbance values for ExVial samples from flies fed media without dye (i.e. background absorbance) at 412, 483, 485, 506, 608 and 630 nm (peak absorbances for Yellow 10, Orange G, Yellow 6, Red A, Green 5, and Light Green SF/Blue 1, respectively) were 0.0009 to 0.0035 ± 0.0003 to 0.0004 (range of mean ± S.D., n = 8 per wavelength). These background absorbances were ignored because they were ~ 100- to 500-fold lower than absorbance values in ExVial samples when flies were fed media containing dyes.

### Coupled CAFE:Con-Ex analyses

Flies were fed 2Y10S3C medium or starved for 18 h, and then provided with a 5% sucrose liquid medium labeled with 1% Orange G, 1% Yellow 10, or no dye. The liquid media were loaded into capillary tubes and provided to flies (10 flies/vial) for 8 h. CAFE and Con-Ex were determined as described^17,18^.

*Ethanol sedation and rapid tolerance:* Assessment of ethanol sedation and rapid tolerance was performed as previously described^10,23–27^. Flies were sorted by sex under brief CO_2_ anesthesia, placed in vials (11 flies/vial) with 2Y10S3C medium without dye or with media labeled with 1% Blue 1, Orange 4, Yellow 10, or Orange G, and allowed to consume media for 24 h in an environmental chamber (25 °C/60% relative humidity). Flies that had consumed media were transferred to empty vials to measure ethanol sedation and tolerance^10,23–27^. Briefly, ST50 (sedation time 50, the time required for 50% of flies in a vial to become sedated) was determined during a first exposure of flies to vapor from 85% ethanol for 60 min. Flies were returned to vials with food (with or without dye consistent with their prior food media) for 4 h, and then a second ST50 was determined. Rapid tolerance was calculated as the ratio of the second ST50 to the first ST50^23,24^.

### Statistical analyses and data reporting

Of the 126 individual data sets generated for this study, 112 data sets (88.9%) passed normal distribution tests (Shapiro–Wilk test, Supplementary Table). We therefore used parametric statistical tests to analyze all data sets reported^20^. Shapiro–Wilk tests for normality, two-tailed t tests, one- and two-way ANOVAs followed by Bonferroni’s multiple comparisons, Pearson correlations and non-linear curve fits were performed with Prism v9.1.1 (GraphPad, San Diego, CA, USA). Power analyses were performed using the average means and standard deviations from several experiments with Orange G and Yellow 10 (see details in legend to Fig. S3) using the on-line calculator available at https://www.stat.ubc.ca/rollin/stats/ssize/n2.html. *p* values and n are reported in the figure legends. Full statistical results and the data used in statistical analyses are reported in the Supplementary Table. Data shown in the figures are mean ± 95% confidence intervals except as noted in figure legends. Individual data points are shown whenever feasible. No data were removed as outliers. *p*-values ≤ 0.05 were considered significant.

## Supplementary Information

Supplementary Information 1.

Supplementary Information 2.

## Data Availability

The datasets generated during and/or analyzed during the current study are available in the Supplementary Table or from the corresponding author on reasonable request.
