# Supplementary material for: Identification of additional dye tracers for measuring solid food intake and food preference via consumption-excretion in Drosophila
Source: Sci Rep. 2022 Apr 13;12:6201. doi: 10.1038/s41598-022-10252-6 (PMC9008003; doi:10.1038/s41598-022-10252-6)
Supplement: Supplementary file 1 — Supplementary Information 1. [file 41598_2022_10252_MOESM1_ESM.pdf]

Supplementary Information

Shell Fig. S1

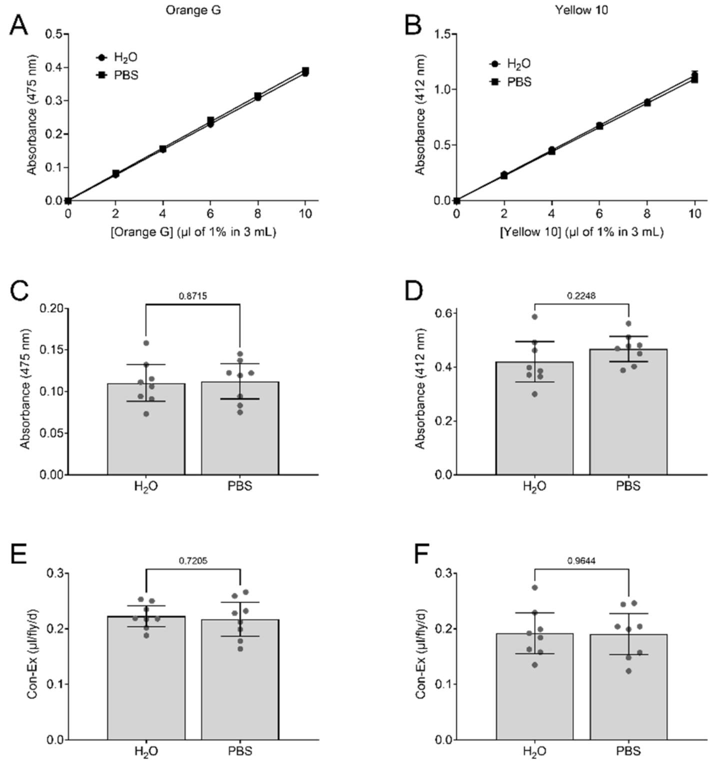

Figure S1. Con-Ex with Orange G and Yellow 10 determined with water and neutral-buffered saline. (A, C and E) Orange G. (B, D and F), Yellow 10. (A, B) Standard curves with Orange G (A) and Yellow 10 (B) in water and phosphate-buffered saline (PBS, pH 7.6). Lines are from best-fit linear regressions (R^2 = 0.9987 to 0.9994; n = 3). (C, D) Absorbance values for ExVial determined with Orange G (C) and Yellow 10 (D) were not significantly different when extracted in water and PBS (t tests, p values shown for pair-wise comparisons, n = 8). (E, F) Con-Ex values determined with Orange G (E) and Yellow 10 (F) were not affected by water versus PBS extraction (t test, p values shown for pair-wise comparisons). Data in panels E and F were derived from data in panels A-D.

Shell Fig. S2

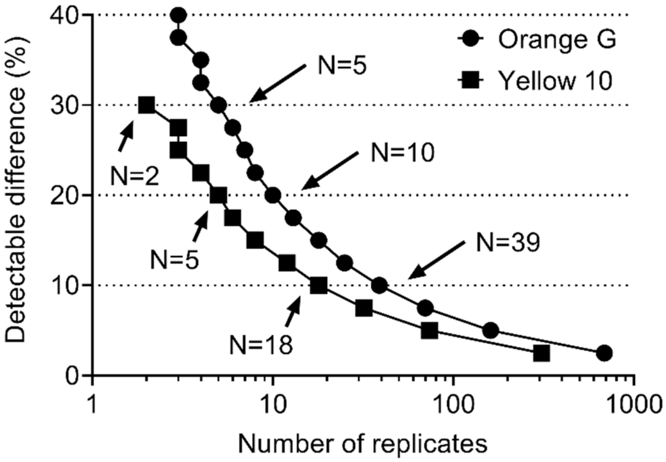

Figure S2. Power analysis of Con-Ex with Orange G and Yellow 10. Power analyses (at 0.8 power and alpha = 0.05) using average mean values for Con-Ex of 0.252 and 0.323 with mean standard deviations of 0.039 and 0.034 µl/fly/d for Orange G and Yellow 10, respectively. Mean values for Orange G were derived from 13 studies shown in Figs. 1-6 and S2. Mean values for Yellow 10 were from 15 studies shown in Figs. 5-6 and S2 as well as Figs. 1-4 in Shell et. al 2018. The numbers of replicates (X-axis) required to detect the indicted differences (Y-axis) are shown.

Shell Fig. S3

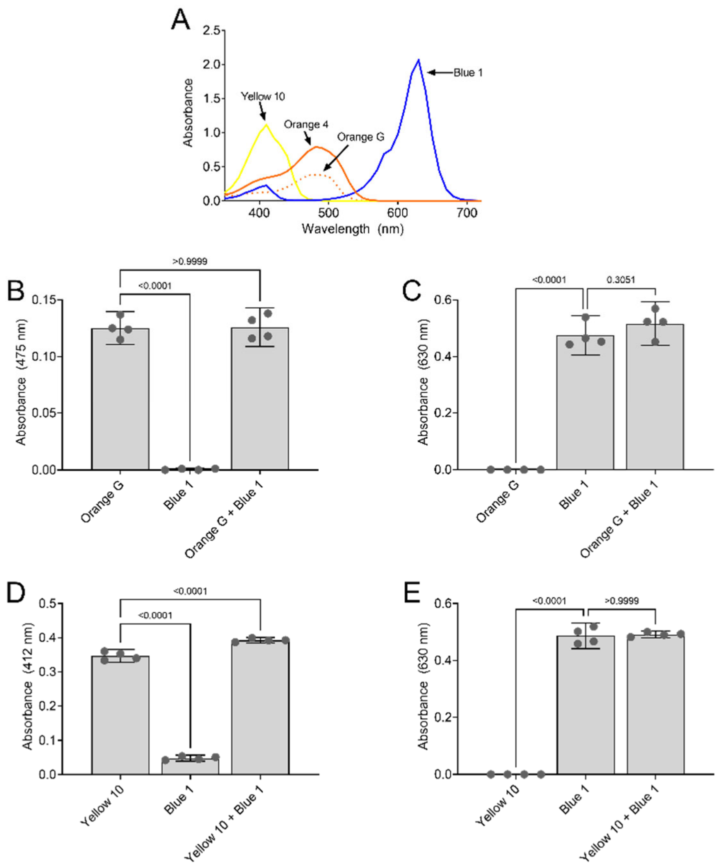

Figure S3. Absorbance spectra of single dyes and absorbance of dye mixtures. (A) Absorbance spectra of the indicated dyes at 0.333% (w/v in water). (B-E) Absorbance of Orange G (0.1%), Blue 1 (0.083%) and a mixture of Orange G plus Blue 1 (B, C), and Yellow 10 (0.1%), Blue 1 (0.083%) and a mixture of Yellow 10 and Blue 1 (D, E) determined at 475 nm (B), 630 nm (C, E) and 412 nm (D). (B-E) The dyes or dye mixtures used had a significant overall effects on absorbance (individual one-way ANOVAs; $p < 0.0001$; $n = 4$). Absorbance at 412 nm of Blue 1 + Yellow 10 was greater than that of Yellow 10 alone (D). Bonferroni's pair-wise comparisons are shown.

Shell Fig. S4

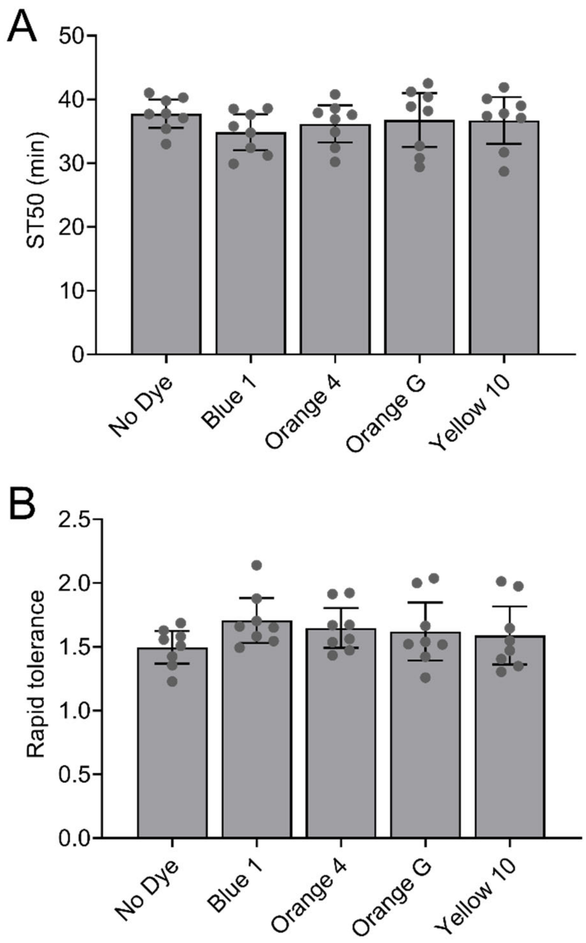

Figure S4. Ethanol sedation and rapid tolerance after consumption of food media labeled with Blue 1, Orange 4, Orange G and Yellow 10. GL females were fed 2Y10S3C food media containing each of the indicated dyes for 24 h. (A) Ethanol sedation. Inclusion of dye in the food media had no effect on ST50 values (one-way ANOVA, $p = 0.6640$, $n = 8$). (B) Rapid tolerance to ethanol. Consumption of media with dye had no effect on rapid tolerance (one-way ANOVA, $p = 0.4361$, $n = 8$).

Supplementary Table. Full statistical results and the data used in statistical analyses in all studies.
